# Association between TP53 rs1042522 gene polymorphism and the risk of malignant bone tumors: a meta-analysis

**DOI:** 10.1042/BSR20181832

**Published:** 2019-03-19

**Authors:** Xin Huang, Fashuai Wu, Zhicai Zhang, Zengwu Shao

**Affiliations:** Department of Orthopaedics, Union Hospital, Tongji Medical College, Huazhong University of Science and Technology, Wuhan 430022, China

**Keywords:** Malignant bone tumor, Polymorphism, TP53

## Abstract

*TP53* is a tumor suppressor gene which is essential for regulating cell division and preventing tumor formation. Several studies have assessed the associations of TP53 single-nucleotide polymorphisms (SNP) with susceptibility of malignant bone tumors, including osteosarcoma and Ewing sarcoma, but the results are inconsistent. In the present meta-analysis, we aimed to elucidate the associations of TP53 rs1042522 genetic polymorphism with the risk of osteosarcoma or Ewing sarcoma. We systematically searched Medline, PubMed, Web of Science, Embase, and the Cochrane Library databases. Eligible studies assessing the polymorphisms in the TP53 rs1042522 gene and risk of malignant bone tumors were incorporated. The pooled odds ratio (OR) with its 95% confidence intervals (95% CIs) were used to assess these possible associations. Five studies with a total of 567 cases and 935 controls were finally included the meta-analysis. Meta-analysis of TP53 rs1042522 polymorphism was significantly associated with an increased risk of malignant bone tumors (G versus C: OR = 1.27, 95% CI 1.08–1.50, *P*=0.005; GG versus GC/CC: OR = 1.55, 95% CI 1.21–2.00, *P*=0.001). Moreover, in a stratified analysis, a statistically significant correlation between this SNP and osteosarcoma risk was also observed. Our results suggest that there are significant associations of TP53 rs1042522 polymorphism with malignant bone tumors risk. More studies based on larger sample sizes and homogeneous samples are warranted to confirm these findings.

## Introduction

Malignant bone tumors are rare human sarcomas and they account for no more than 0.2% of all kinds of tumors [1]. Osteosarcoma and Ewing sarcoma are the most common malignant bone tumors in children and adolescents [2]. Despite great advances in the tumor treatments, the overall survival for osteosarcoma or Ewing sarcoma patients is still unsatisfactory [3]. It is known to all that both osteosarcoma and Ewing sarcoma are complex and multifactorial diseases, and the carcinogenesis of those malignant bone tumors is still uncertain [4]. However, it is very likely that there are gene–environment interactions in the carcinogenesis of malignant bone tumors, and the genetic susceptibility factors play a vital role in the development of osteosarcoma or Ewing sarcoma [5,6].

*TP53* gene encodes a tumor suppressor protein p53, which is essential for cell cycle regulation and plays an important role in cancer prevention through regulating apoptosis, genomic stability, and inhibition of angiogenesis [7,8]. And recent studies show that the p53 expression level can be altered by the genetic polymorphisms in the *TP53* gene [9]. TP53 rs1042522 polymorphism is one of the most known polymorphisms of TP53 and it is the single nucleotide polymorphism (SNP) at codon 72, located at the exon 4 of this gene. This SNP is a non-conservative change of the wild-type variants Arginine (CGC) and Proline (CCC) (Arg72Pro–dbSNP ID: rs1042522), that results in different biological functions of p53 [10].

There are several studies published to assess the associations of TP53 rs1042522 genetic polymorphisms with risk of osteosarcoma or Ewing sarcoma [11–14]. Four of the studies are about osteosarcoma and two of them are about Ewing sarcoma. The studies reported contradictory results and failed to confirm a strong and consistent association. In Wang’s [15] publication, just two of the included studies, with a total sample size of 410 osteosarcoma patients and 470 controls, are about associations between TP53 rs1042522 gene polymorphism and osteosarcoma risk. The studies above are limited in discrete outcome and sample size, making the results not credible enough. Thus, we conducted a meta-analysis of epidemiological studies with a larger sample size to shed some light on the associations of TP53 genetic polymorphisms with risk of malignant bone tumors comprising osteosarcoma and Ewing sarcoma. As a part of our analysis, stratified analysis according to different types of malignant bone tumors and ethnicity were also conducted.

## Materials and methods

### Search strategy and eligibility criteria

A computerized literature search was performed in the Medline, PubMed, Web of Science, and Embase databases. The search strategy included the terms (‘bone tumor’ or ‘osteosarcoma’ or ‘Ewing sarcoma’) and (‘P53’ or ‘TP53’ or ‘rs1042522’). To be eligible for inclusion in the meta-analysis, a study must meet the following criteria: (i) case–control study or cohort study, (ii) identification of malignant bone tumors which was confirmed histologically or pathologically, (iii) having an available genotype or allele frequency for estimating an odds ratio (OR) with 95% confidence interval (95% CI) or hazard ratio (HR) with 95% CI, (iv) genotype frequencies in controls were consistent with those expected from Hardy–Weinberg equilibrium (*P*>0.05). Whereas, case reports, reviews, and studies containing overlapping data were excluded.

### Data extraction and quality assessment

Two investigators (X.H. and F.W.) evaluated the eligibility of all retrieved studies and extracted the relevant data independently. Extracted databases were then cross-checked between the two authors to rule out any discrepancy. Disagreement was resolved by consulting with a third investigator (Z.Z.). The following data of each eligible study were extracted independently: name of first author, year of publication, countries, ethnicity, genotype or allele frequencies of TP53 rs1042522 polymorphisms, and OR with its 95% CI. The study quality was assessed in accordance with the Newcastle–Ottawa Scale (NOS). Eight items were extracted, and each item scored 1. The total scores ranged from 0 to 8. If the scores were ≥7, then the study was considered high quality.

### Statistical analysis

The statistical analysis was performed using STATA 14. Estimates were summarized as ORs with 95% CIs for each study. The between-study heterogeneity was evaluated by using the chi-square test and the *I^2^* statistic. An *I^2^* value of >50% of the *I^2^* statistic was considered to indicate significant heterogeneity [16]. When a significant heterogeneity existed across the included studies, a random-effects model was used for the analysis. Otherwise, the fixed-effects model was used. Subgroup analyses were performed to detect the source of heterogeneity. We further conducted sensitivity analyses to substantiate the stability of results and detect the potential source of heterogeneity. Publication bias was evaluated qualitatively by inspecting funnel plots and quantitatively through the Begg’s and Egger’s tests. A two-tailed *P*-value <0.05 implied a statistically significant publication bias.

## Results

### Search results

**Figure 1:**
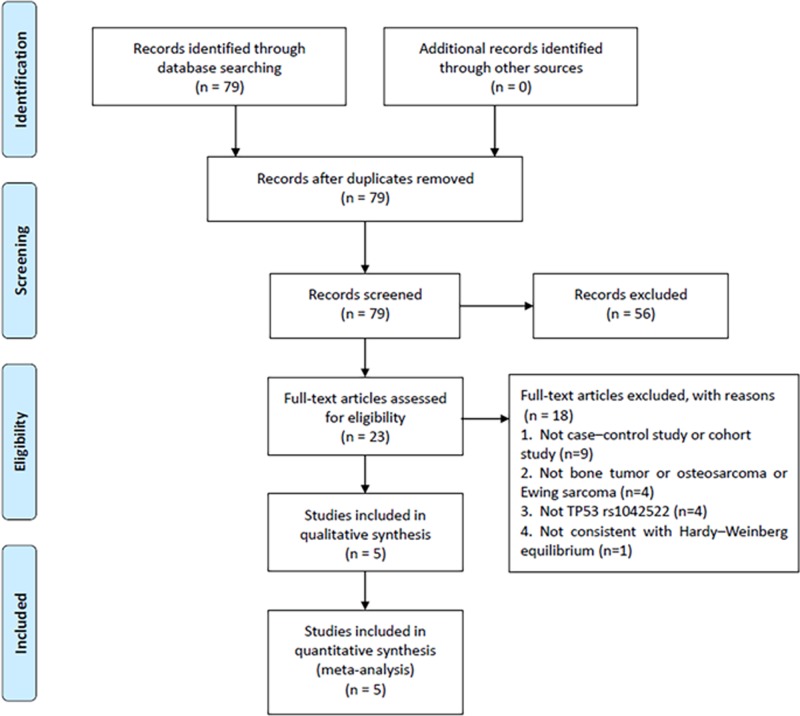
Flow chart of the study selection process

The study selection process is illustrated in Figure 1. A total of 79 potential articles were identified from the databases search. Amongst these articles, 56 were excluded after abstract review, leaving 23 articles for the full-text review. In the review, 18 studies were excluded for the reasons as follows: nine were eliminated because they were neither case–control study or cohort study, four were irrelevant to bone tumor or osteosarcoma or Ewing sarcoma, four studies were not studies on the role of TP53 rs1042522 on patient survival or disease progression, one was not consistent with Hardy–Weinberg equilibrium. Finally, five studies with a total of 567 cases and 935 controls that met the inclusion criteria were included in this meta-analysis.

### Study selection and characteristics

Baseline characteristics of the included studies are presented in Table 1. Amongst these eligible studies, Toffoli et al. [11] and Hattinger et al. [12] investigated the role of TP53 rs1042522 in osteosarcoma development amongst Caucasians. In addition, Ru et al. [13] examined effects of this SNP on the risk of osteosarcoma amongst Chinese, and Thurow et al. [] for Ewing sarcoma in mixed populations. Otherwise, the risk of both osteosarcoma and Ewing sarcoma amongst Caucasians was assessed by Ito et al. [14]. The publication years of the eligible studies ranged from 2009 to 2016. Importantly, genotype frequencies in controls were consistent with those expected from Hardy–Weinberg equilibrium (*P*>0.05). The NOS confirmed that all the studies were of high quality (Supplementary Table S1).

**Table 1:** Main characteristics of the studies included in this meta-analysis

Study ID	SNP	Year	Country	Disease	Ethnicity	Case group			Control group			*P* for HWE	Quality
						CC	CG	GG	CC	CG	GG		
**TP53**													
Ru	rs1042522	2015	China	OS	Chinese	59	106	44	162	194	64	0.637	Y
Toffoli	rs1042522	2009	Italy	OS	Caucasian	16	43	142	17	87	146	0.296	Y
Hattinger	rs1042522	2016	Italy	OS	Caucasian	71	21	8	59	34	7	0.497	Y
Thurow	rs1042522	2013	Brazil	EWS	Mixed	2	8	14	8	43	40	0.454	Y
Ito	rs1042522	2011	Australia	OS	Caucasian	0	6	11	3	14	20	0.804	Y
Ito	rs1042522	2011	Australia	EWS	Caucasian	2	7	7	3	14	20	0.804	Y

Abbreviations: EWS, Ewing sarcoma; HWE, Hardy–Weinberg equilibrium; OS, osteosarcoma; Y, yes.

### Meta-analysis results

**Figure 2:**
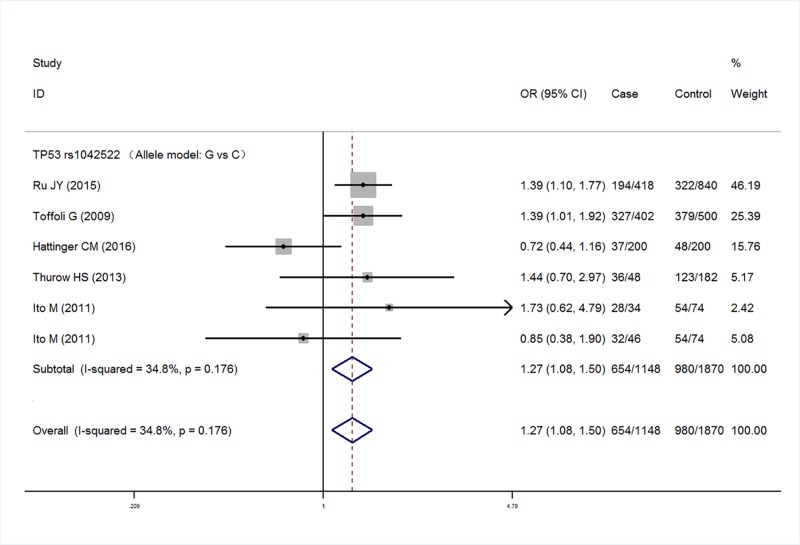
Meta-analysis for TP53 rs1042522 polymorphism (G versus C) in malignant bone tumors

**Figure 3:**
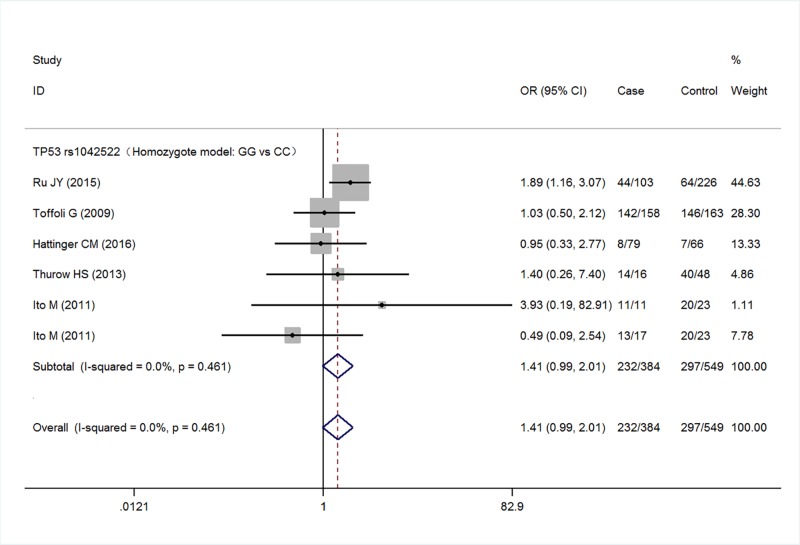
Meta-analysis for TP53 rs1042522 polymorphism (GG versus CC) in malignant bone tumors

**Figure 4:**
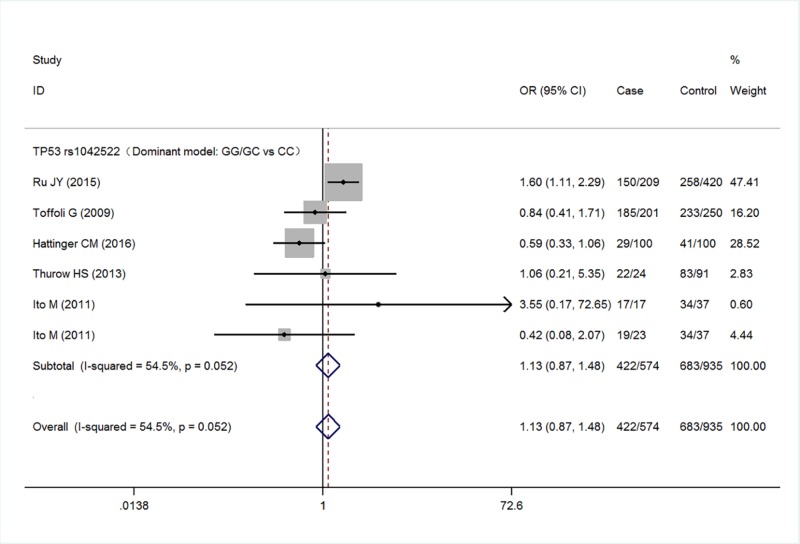
Meta-analysis for TP53 rs1042522 polymorphism (GG/GC versus CC) in malignant bone tumors

**Figure 5:**
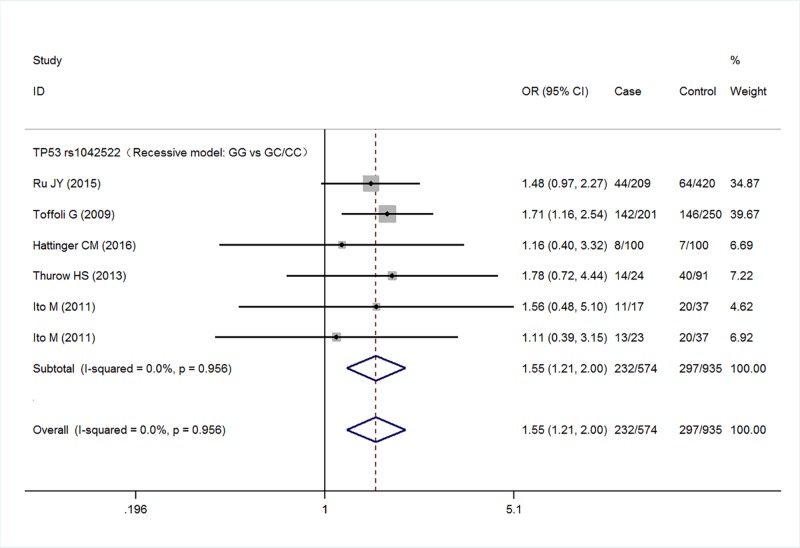
Meta-analysis for TP53 rs1042522 polymorphism (GG versus GC/CC) in malignant bone tumors

Five studies with a total of 567 cases and 935 controls were finally included the meta-analysis. Meta-analysis of TP53 rs1042522 polymorphism was associated with an increased risk of malignant bone tumors (G versus C: OR = 1.27, 95% CI 1.08–1.50, *P*=0.005; GG versus CC: OR = 1.41, 95% CI 0.99–2.01, *P*=0.057; GG/GC versus CC: OR = 1.13, 95% CI 0.87–1.48, *P*=0.365; GG versus GC/CC: OR = 1.55, 95% CI 1.21–2.00, *P*=0.001) (Figures 2–5 respectively). And the association was statistically significant under allele model (G versus C) and recessive model (GG versus GC/CC) (*P*<0.05) .

Subgroup analysis was conducted on the association between TP53 rs1042522 polymorphism and the risk of malignant bone tumors (Table 2). After stratifying by ethnicity, there were four studies including patients amongst Caucasians (GG versus GC/CC: OR = 1.565, 95% CI 1.121–2.184, *P*=0.009) and apparently no heterogeneity was obtained (*P*=0.814, *I^2^* = 0.0%). Because only two studies assessed the patients amongst non-Caucasians, we did not perform the meta-analysis. Stratified analysis according to disease was also conducted. For osteosarcoma, there were four studies including 527 cases and 807 controls, and no between-study heterogeneity was found (*P*=0.900, *I^2^* = 0.0%). In fixed-effect model, a statistically significant correlation between the TP53 rs1042522 polymorphism and osteosarcoma risk was observed (GG versus GC/CC: OR = 1.569, 95% CI 1.196–2.057, *P*=0.001). For Ewing sarcoma, TP53 rs1042522 polymorphism was associated with an increased risk of Ewing sarcoma (GG versus GC/CC: OR = 1.452, 95% CI 0.732–2.882, *P*=0.262). However, the association was not statistically significant (*P*>0.05).

**Table 2:** Subgroup analysis of association between TP53 rs1042522 SNP and malignant bone tumors

rs1042522	OR	95% CI		*P*-value	*l^2^*	*P* for heterogeneity
**Ethnicity**						
**Caucasian**						
G versus C	1.134	0.887	1.448	0.315	51.0	0.106
GG versus CC	0.991	0.574	1.709	0.973	0.0	0.680
GG/GC versus CC	0.692	0.452	1.058	0.089	0.0	0.552
GG versus GC/CC	1.565	1.121	2.184	0.009	0.0	0.814
**Disease**						
**Osteosarcoma**						
G versus C	1.284	1.078	1.528	0.005	54.4	0.087
GG versus CC	1.494	1.029	2.169	0.035	0.0	0.397
GG/GC versus CC	1.167	0.887	1.537	0.270	68.3	0.024
GG versus GC/CC	1.569	1.196	2.057	0.001	0.0	0.900
**Ewing sarcoma**						
G versus C	1.146	0.671	1.955	0.618	0.0	0.338
GG versus CC	0.838	0.272	2.584	0.759	0.0	0.378
GG/GC versus CC	0.669	0.224	1.996	0.471	0.0	0.424
GG versus GC/CC	1.452	0.732	2.882	0.286	0.0	0.498

### Publication bias and sensitivity analysis

The funnel plot did not indicate any evidence of publication bias in this analysis (Supplementary Figure S1). No evidence of publication bias was observed from Begg’s funnel plot (*P*=0.452) (Supplementary Figure S2) and Egger’s test (*P*=0.923) (Supplementary Figure S3). To sum up, the possibility of publication bias could be excluded. The sensitivity analysis showed that the results of the meta-analysis did not change when studies were omitted one by one (Supplementary Figure S4) .

## Discussion

TP53 plays an important role in the carcinogenesis of various cancers including osteosarcoma or Ewing sarcoma. There were several studies assessing the effects of TP53 polymorphisms on the risk of malignant bone tumors, but there was no comprehensive assessment of the effects and the studies reported different results. For example, no associations of the TP53 Arg72Pro SNP (TP53 rs1042522) with Ewing sarcoma were found in Thurow et al.’s [] article. No increased or decreased risk to develop osteosarcoma was observed in association with the Pro/Pro genotype variant in Toffoli et al.’s [11] research. Hattinger et al. [12] found that TP53 Arg72Pro SNP (TP53 rs1042522) significantly associated with increased relative risk to develop osteosarcoma. Ru et al. [13] demonstrated that subjects with the GG genotype of rs1042522 had significantly increased osteosarcoma risk (OR = 1.89, 95% CI: 1.16–3.07) compared with those who carrying the CC genotype. And in Ito et al.’s [14] article, no statistically significant difference in the polymorphism frequency were found between benign and malignant tumor groups including osteosarcoma and Ewing sarcoma (R72P, *P*=0.958)]. Here upon, we comprehensively searched the up-to-date electronic databases and enrolled five independent case–control studies with a total of 567 cases and 935 controls into our meta-analysis to reveal the associations between TP53 genetic polymorphisms and risk of malignant bone tumors. There were four studies on the association between this SNP and risk of osteosarcoma, and two studies for the risk of Ewing sarcoma. The results of our analysis showed that TP53 rs1042522 polymorphism significantly increased the risk of malignant bone tumors.

The p53/MDM pathway comprises alternative reading frame (ARF), murine double minute 2 (MDM2), MDM4, and p53 proteins. Classically, MDM2 (or MDM4) could bind p53 and then promote proteasomal degradation of the tumor suppressor [17]. The TP53 protein which is central for maintaining genomic stability and preventing tumor formation is best characterized as a DNA-binding transcription factor with potential to bind to several hundred different promoter elements in the genome, hence regulating expression of hundreds of genes involved in control of processes related to tumor growth, including cell cycle regulation, DNA preservation, apoptosis, angiogenesis inhibition, and cellular senescence [18–20]. Its coding gene *TP53* is highly mutated in ∼50% of human cancers [21,22]. The loss of p53 function by mutations in *TP53* gene or in genes of proteins that interact with p53 protein ablates its ability to prevent tumor formation and favors cellular proliferation and tumor initiation and progression. The *TP53* gene mutation has been observed in the classic Li-Fraumeni Syndrome [23,24] including multiple tumors [25–27]. Although osteosarcoma is commonly observed in this syndrome, not all individuals with TP53 gene variants can develop osteosarcoma. Twelve genetic variations in TP53 have been studied to indicate a link between TP53 polymorphisms and osteosarcoma risk [28]. Amongst those variations in TP53, the Arg72Pro SNP is the most commonly studied mutation amongst Caucasians population.

In our study, after stratifying by ethnicity, we observed in the meta-analysis that OR values amongst Caucasian populations were consistent with our total populations, suggesting that the existence of population differences could lead to the same SNP effects. Because only two studies assessed the patients amongst non-Caucasians population, we failed to perform further meta-analysis. Two major malignant bone tumors are osteosarcoma and Ewing sarcoma. In our meta-analysis, four studies with a total of 527 cases and 807 controls assessed the risk of osteosarcoma. Results showed that TP53 rs1042522 polymorphism was significantly associated with an increased risk of osteosarcoma. However, no statistically significant correlation between the TP53 rs1042522 polymorphism and Ewing sarcoma risk was observed. Thurow et al. [10] genotyped the TP53 Arg72Pro SNP in 24 Ewing sarcoma patients and 91 control individuals and the small sample size may account for the no statistically significant correlation.

Taken all results into consideration, the present meta-analysis has several strengths. First, we used a comprehensive search strategy, and had a well-defined inclusion and exclusion criteria. Second, two reviewers performed the study selection and extracted data independently, and discrepancies were resolved by consensus. Third, we assessed the quality of the included studies by predefined criteria and the score of included studies was high. Finally, all genotype data extracted from the studies were reported in the study.

Nevertheless, there are still some limitations. First, the number of cases is still relatively small; which may be inherent to the low incidence of osteosarcoma or Ewing sarcoma. And the small number of only five studies indicated that the statistical power to detect differences was suboptimal. However, the pooled results in our review were more reliable than the results in each of the individual studies. Second, the heterogeneity of the studies was high in the case of homozygote model (GG versus CC) and dominant model (GG/GC versus CC). We explored the sources of heterogeneity by sensitivity analysis. However, none of those studies altered the pooled OR significantly. And we further performed subgroup analysis and showed that ethnicity or disease may account for the heterogeneity. Third, only two studies assessed the patients amongst non-Caucasians. Therefore, additional studies with other ethnic populations are warranted to assess the association between TP53 rs1042522 polymorphism and the risk of malignant bone tumors. Fourth, malignant bone tumor comprises osteosarcoma, Ewing sarcoma, chondrosarcoma and synovial sarcoma, and so on. Osteosarcoma and Ewing’s sarcoma are the two most common primary malignant bone tumors in children, adolescents, and young adults, which cause serious damage to human health. In our research, considering the degree of malignancy, population of high incidence and bad prognosis of these two tumors are similar, we mainly concentrate on osteosarcoma and Ewing’s sarcoma and combine them together to be analyzed. However, there are many differences between these two types of tumors, such as histological origin, development, tumor location, and standard treatment strategy, which may cause heterogeneity and influence the reliability of our results in some degree. Fifth, the majority of the included trials mainly reported data on rs1042522 polymorphism, thus we were unable to examine the association of other SNPs of the *TP53* gene with malignant bone tumors risk. Finally, the web resources such as the 1000 Genomes Browser providing the allele frequencies of TP53 rs1042522 polymorphism amongst different main populations were not considered and utilized in our research. The allele frequencies were significantly different with the values calculated from our included studies. All the limitations mentioned above might lead to false-positive findings. Therefore, additional prospective studies with larger sample sizes including participants of other ethnic populations are warranted. Besides, reasonable utilization of the web resources containing TP53 rs1042522 allele frequencies of different population might be considered to enlarge our control groups and provide us a significant extension of our finding in the future.

## Conclusion

In conclusion, the present meta-analysis indicates that TP53 rs1042522 polymorphism is the genetic risk factor for the susceptibility of malignant bone tumors. Well-designed studies with larger sample sizes and various SNPs are warranted to confirm our current findings.

## Supporting information

**Figure S1:** Funnel plot for TP53 rs1042522 polymorphism in malignant bone tumors.

**Figure S2:** Begg’s funnel plot for TP53 rs1042522 polymorphism in malignant bone tumors.

**Figure S3:** Egger’s funnel plot for TP53 rs1042522 polymorphism in malignant bone tumors.

**Figure S4:** Sensitivity analysis for TP53 rs1042522 polymorphism in malignant bone tumors.

**Supplementary Table 1:** Quality assessment of eligible studies (Newcastle-Ottawa Scale).

## Abbreviations

MDM: murine double minute
NOS: Newcastle–Ottawa Scale
OR: odds ratio
SNP: single-nucleotide polymorphism
95% CI: 95% confidence interval
