# Supplementary material for: Association between TP53 rs1042522 gene polymorphism and the risk of malignant bone tumors: a meta-analysis
Source: Biosci Rep. 2019 Mar 19;39(3):BSR20181832. doi: 10.1042/BSR20181832 (PMC6422898; doi:10.1042/BSR20181832)
Supplement: Supplementary file 1 [file bsr-39-bsr20181832_Supp1.pdf]

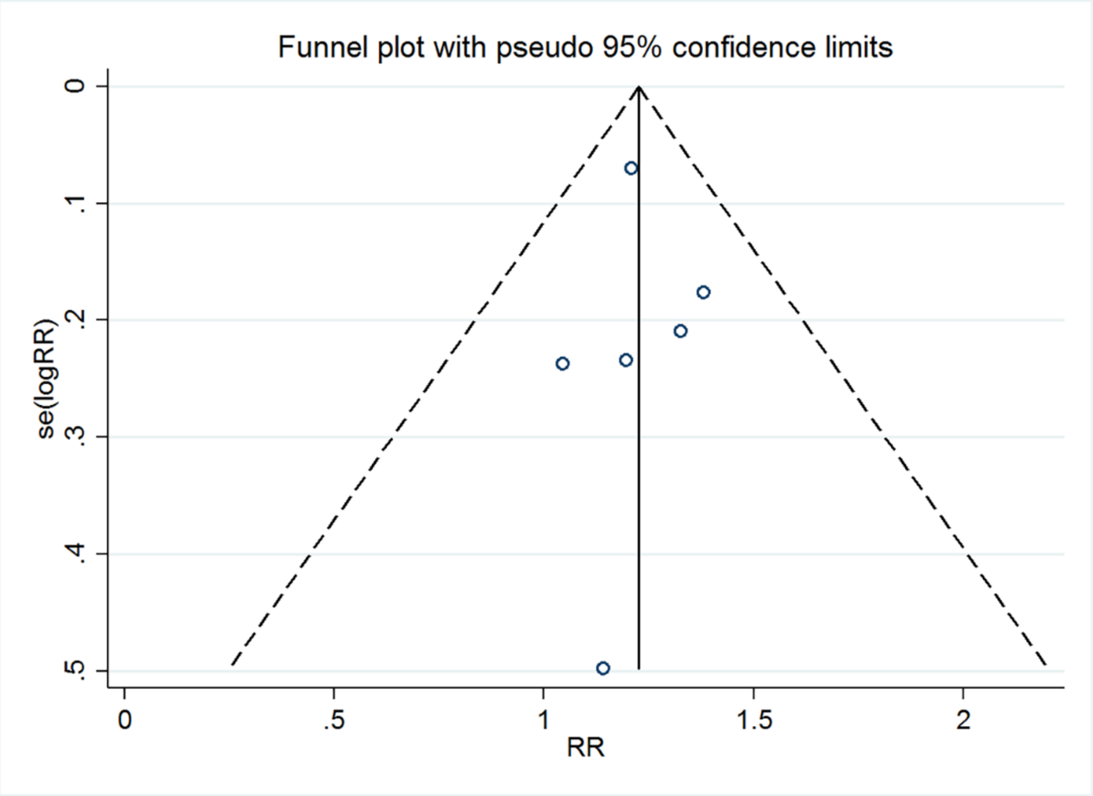

Figure S1. Funnel plot for TP53 rs1042522 polymorphism in malignant bone tumors.

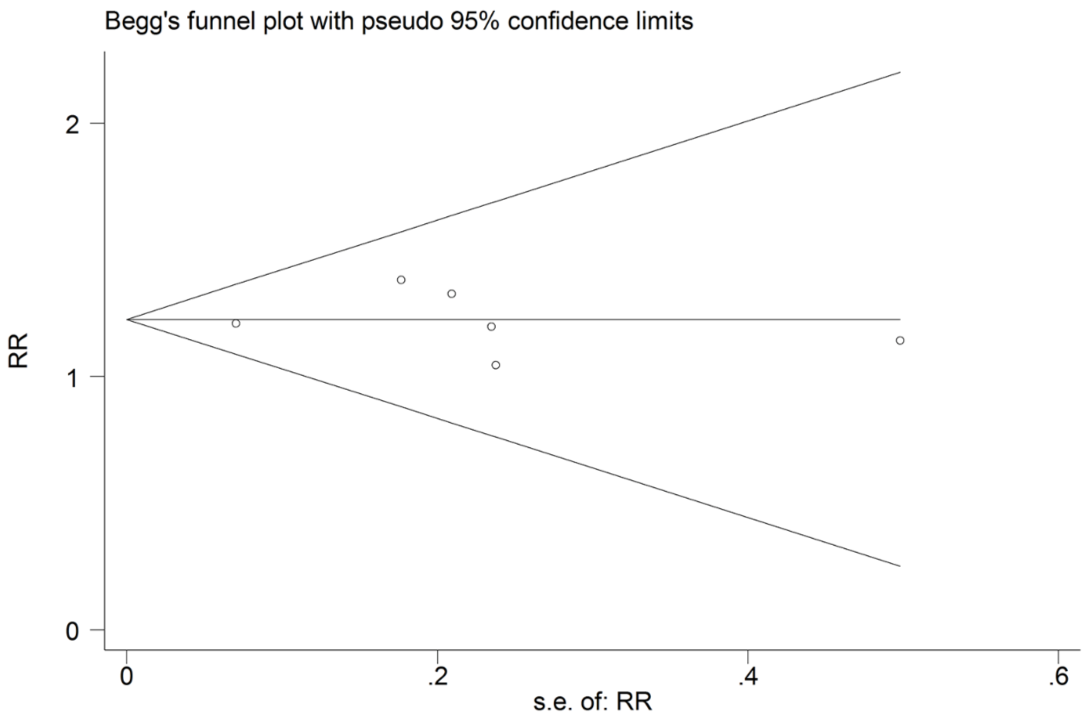

Figure S2. Begg’s funnel plot for TP53 rs1042522 polymorphism in malignant bone tumors.

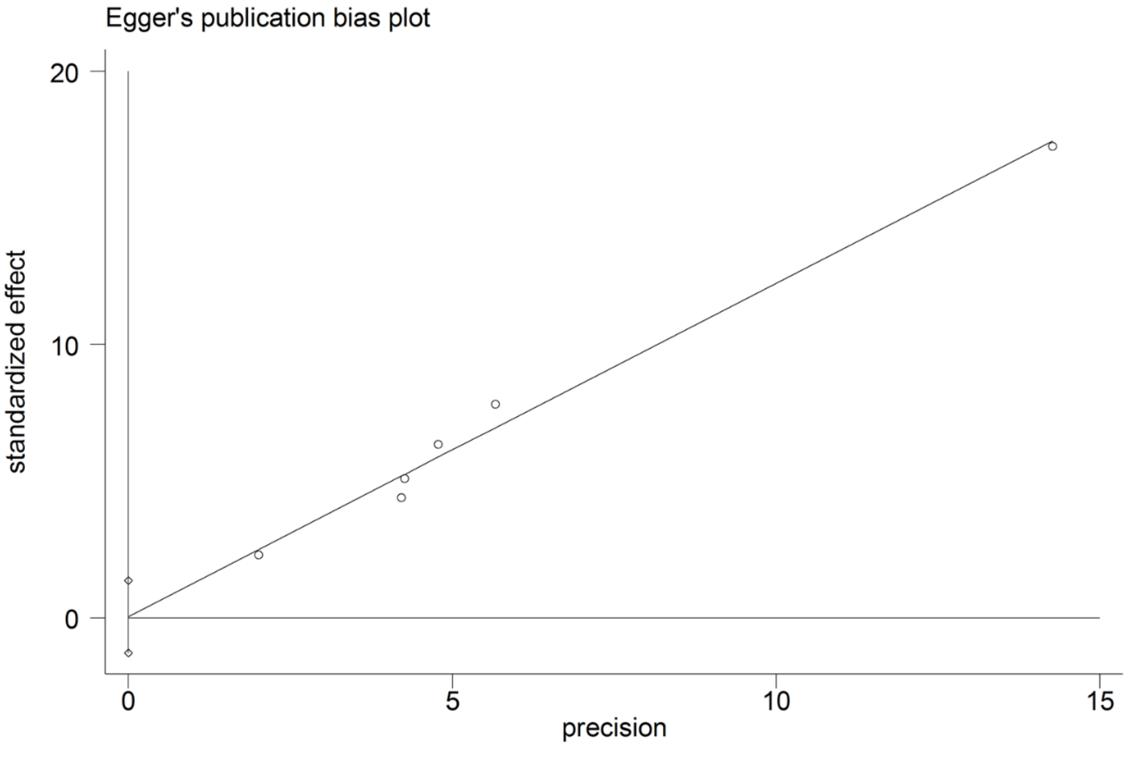

Egger's publication bias plot
standardized effect
precision
20
10
0
0
5
10
15

Figure S3. Egger’s funnel plot for TP53 rs1042522 polymorphism in malignant bone tumors.

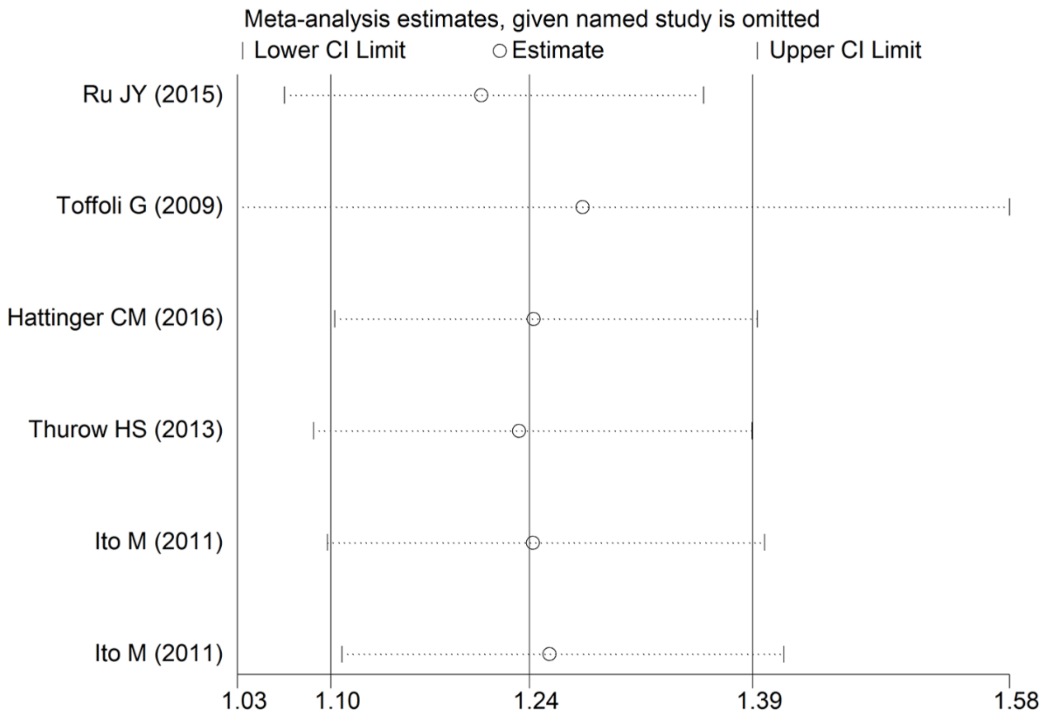

Figure S4. Sensitivity analysis for TP53 rs1042522 polymorphism in malignant bone tumors.

Supplementary Table 1. Quality assessment of eligible studies (Newcastle-Ottawa Scale).

**Study**	**Selection**				**Comparability**		**Outcome**		**Total**
	**Adequacy of case definition**	**Number of case**	**Representativeness of the cases**	**Ascertainment of exposure**	**Ascertainment of detection method**	**Ascertainment of cut-off**	**Assessment of outcome**	**Adequate follow up**	
Ru 2015	1	1	1	1	1	1	1	1	8
Toffoli 2009	1	1	1	1	1	1	1	1	8
Hattinger 2016	1	1	1	1	1	1	1	1	8
Thurow 2013	1	1	1	1	1	1	1	0	7
Ito 2011	1	1	1	1	1	1	1	0	7
